# Bone grafts and synthetic substitutes in dental applications: a comprehensive review of molecular mechanisms, materials evolution, and clinical perspective

**DOI:** 10.3389/fbioe.2025.1759864

**Published:** 2026-01-12

**Authors:** Huachun Wang, Jingyang Kang

**Affiliations:** Department of Stomatology, Qilu Hospital (Qingdao), Cheeloo College of Medicine, Shandong University, Qingdao, China

**Keywords:** biomaterials, bone graft, bone regeneration, dental materials, synthetic bone substitute

## Abstract

Bone grafting plays a critical role in oral and maxillofacial surgery by restoring structural integrity and function in patients with bone defects resulting from congenital anomalies, trauma, tumor resection, or periodontal disease. To meet clinical needs, various types of bone grafts and substitutes have been utilized, including autografts, allografts, xenografts, and synthetic materials. The success of these materials depends on their ability to support bone regeneration through key biological and mechanical functions. Bone is a hierarchically organized tissue that undergoes continuous remodeling, and effective graft materials must integrate osteogenic cells, osteoinductive signals, osteoconductive scaffolds, mechanical stability, vascularization, and a favorable host environment. While autografts remain the gold standard, limitations such as donor site morbidity and limited availability have led to increased use of alternative materials. Synthetic substitutes offer advantages in customization and availability but often require enhancement to improve biological performance. Recent strategies such as three-dimensional printing, incorporation of growth factors, and nanotechnology-enabled delivery systems are being explored to create next-generation graft materials. This review provides a comprehensive overview of the structural and biological principles underlying bone regeneration, the historical and conceptual evolution of grafting strategies, and the advantages and limitations of current materials used in oral and maxillofacial reconstruction. periodontal disease.

## 1 Introduction

Bone grafting is a fundamental procedure in oral and maxillofacial surgery, widely employed to restore the structure and function of the jaws and craniofacial skeleton. It plays a critical role in the management of various clinical conditions, including congenital anomalies, traumatic bone defects, tumor resection sites, and alveolar bone loss caused by periodontal disease (Usui et al., 2021; Yu and Wang, 2000; Major et al., 2020; Titsinides et al., 2019). These conditions often lead not only to aesthetic impairments but also to compromised oral functions such as mastication, articulation, and respiration.

To address these challenges, a wide range of bone grafts and substitutes have been developed. Bone grafts are biologically derived materials typically classified into three main types: autografts, allografts, and xenografts (Titsinides et al., 2019). These natural grafts differ in their biological properties, availability, and clinical applicability. In addition, bone substitutes refer to synthetic, inorganic, or bioorganic composites designed to replace autologous or allogeneic bone in the treatment of bone defects (Pryor et al., 2009). Among these, synthetic substitutes, commonly referred to as alloplastic materials, have attracted increasing attention due to their controlled composition, reproducibility, and absence of disease transmission risk.

However, the rational design and functional evaluation of these materials require a deep understanding of native bone structure and the physiological principles of remodeling. Successful grafting strategies must mimic the natural healing environment, a framework conceptualized as the “diamond concept” of bone healing (Giannoudis et al., 2007). This framework outlines the necessity of integrating essential biological elements such as osteogenic cells, osteoinductive signals, and vascularization to achieve optimal reconstruction (Marie and Cohen-Solal, 2018).

An ideal bone graft or substitute must exhibit excellent biocompatibility, support cell adhesion and differentiation, possess a porous structure to enable vascular infiltration, and display mechanical properties comparable to native bone (Haugen et al., 2019; Wang and Yeung, 2017; Bhatt and Rozental, 2012). Additionally, the material should degrade gradually and be replaced by new bone of equivalent quality. To meet diverse clinical demands, various grafting materials have been developed. Autografts remain the clinical gold standard due to their intrinsic osteogenic, osteoinductive, and osteoconductive properties (Misch, 2010). However, limitations such as restricted availability and donor site morbidity have prompted the use of alternatives. Allografts and xenografts offer structural support but lack viable cells and may trigger immune responses or present mechanical shortcomings (Miron, 2000). Synthetic substitutes such as calcium phosphate ceramics, bioactive glass, and biodegradable polymers provide customizable architectures and avoid donor-related complications, but they often require biological enhancement to improve their performance (Sakdejayont et al., 2025; Pryor et al., 2009).

Recent advances such as three-dimensional printing, computer-aided design, and controlled delivery of bioactive molecules are being investigated to improve graft integration and functionality (Abdelaziz et al., 2023; Percival et al., 2024; Koons et al., 2020). These approaches aim to create next-generation materials that not only replicate the structural features of native bone but also interact dynamically with the surrounding biological environment. A detailed understanding of these design principles, biological interactions, and clinical outcomes is essential for the continued advancement of bone grafting technologies in oral and maxillofacial reconstruction.

This review aims to provide a comprehensive overview of bone grafts and synthetic substitutes used in dental applications, summarizing their molecular mechanisms, material evolution, clinical performance, and the advantages and limitations of each approach.

## 2 Materials and methods

An electronic search was conducted in PubMed, Scopus, and Web of Science up to October 2025, supplemented by reference list screening of key publications. The review followed a structured process inspired by the PRISMA ScR guidelines. Eligible studies were English language, peer reviewed *in vitro*, *in vivo*, and clinical research focusing on autografts, allografts, xenografts, or synthetic substitutes in oral and maxillofacial applications. From the initial search, relevant evidence on the biological basis of bone regeneration, including osteogenesis, osteoinduction, osteoconduction, mechanical stability, vascularization, and host graft interactions, was identified. Data were extracted and summarized for material type, structural and biological properties, application strategies, and reported outcomes, with particular attention to both natural grafts and synthetic materials as well as emerging technologies aimed at improving clinical performance.

## 3 Bone biology and remodeling

### 3.1 Skeletal structure and composition

**FIGURE 1:**
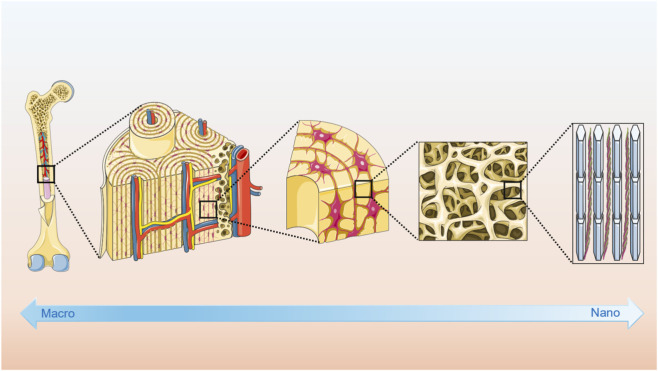
Hierarchical structure of bone from macro to nanoscale.

Bone is a structurally complex and hierarchically organized tissue, with architecture spanning from the organ level to the molecular and atomic scale (Hamandi and Goswami, 2022; Koushik et al., 2023; Reznikov et al., 2018). As illustrated in Figure 1, this multiscale organization begins at the macroscopic level with the distinction between cortical bone, which forms the dense outer layer and provides mechanical support, and cancellous bone, which forms a porous internal network involved in metabolic exchange and rapid remodeling. Cortical bone is composed of osteons, concentric lamellar structures surrounding vascular channels, while cancellous bone contains lamellae that are arranged more parallel to each other (Isojima and Sims, 2021).

At the microscopic level, the bone matrix is primarily composed of type I collagen fibers, formed by two α1 chains and one α2 chain, accounting for approximately 90 percent of the organic component. In lamellar bone, these fibers are organized into arched structures to maximize collagen density within a given volume. Hydroxyapatite crystals with the formula Ca_10_(PO_4_)_6_(OH)_2_ are deposited both within and around the collagen fibers, and their orientation is generally aligned with the direction of the fibrils. This composite of collagen and mineral provides bone with its unique combination of tensile strength and compressive stiffness (Buss et al., 2022; Wagermaier et al., 2006).

At the nanoscale, collagen molecules self-assemble into triple helices and further into microfibrils, which associate with aligned hydroxyapatite nanocrystals to form mineralized collagen fibers. These fibers are considered the fundamental structural units of bone. Their orderly arrangement into lamellae and higher-level structures ensures the mechanical integrity, load-bearing function, and adaptive remodeling capacity of the skeleton throughout life (Zimmermann and Ritchie, 2015; Tertuliano and Greer, 2016).

### 3.2 Bone formation and remodeling

**FIGURE 2:**
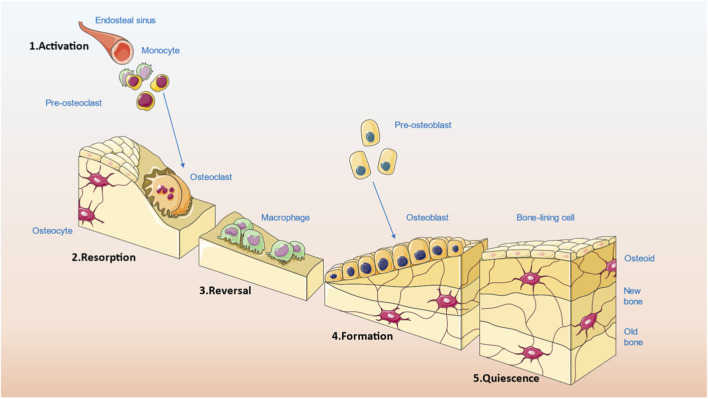
The bone remodeling cycle and its cellular dynamics.

Bone remodeling is a tightly regulated physiological process that maintains skeletal integrity and mineral homeostasis through the sequential activity of specialized cells (Schmidt, 2021). As illustrated in Figure 2, the remodeling cycle consists of five phases: activation, resorption, reversal, formation, and quiescence (Koushik et al., 2023; Tonna et al., 2014). The process begins with the recruitment of circulating monocytes, which differentiate into pre-osteoclasts and subsequently fuse to form mature osteoclasts. These cells degrade mineralized bone matrix, generating resorption cavities (Lin et al., 2020).

Following resorption, the reversal phase prepares the bone surface for subsequent formation. This transitional phase involves the presence of various mononuclear cells, including monocytes, osteocytes released from the matrix, and pre-osteoblasts. Although the molecular mechanisms linking resorption and formation remain incompletely defined, several matrix-derived signaling factors have been proposed as coupling agents. These include transforming growth factor-beta (TGF-β), insulin-like growth factors (IGF), bone morphogenetic proteins (BMPs), platelet-derived growth factor (PDGF), and fibroblast growth factors (FGFs) (Tang et al., 2009; Siddiqui and Partridge, 2016; Behr et al., 2010; Kim et al., 2012; Cassuto et al., 2025).

During the formation phase, pre-osteoblasts are recruited and proliferate in response to signals released from the resorbed matrix. They differentiate into osteoblasts, which secrete osteoid that fills the resorption lacunae and initiates new bone deposition (Rucci, 2008). A portion of these osteoblasts becomes embedded in the matrix as osteocytes, while others flatten and remain at the surface as bone-lining cells. These cells play a role in maintaining the integrity of the newly formed bone and remain in contact with deeper osteocytes via cellular extensions (Šromová et al., 2023).

Mineralization of the osteoid typically begins around 30 days after its deposition and is completed by approximately 90 days in trabecular bone and 130 days in cortical bone (Patel and Saxena, 2025). This is followed by a quiescent phase during which the bone surface remains stable until the next remodeling cycle is initiated. Under normal conditions, the amount of bone formed during each cycle is balanced with the amount resorbed, ensuring long-term skeletal homeostasis (Bolamperti et al., 2022).

## 4 Biological principles and conceptual models

### 4.1 Historical development of bone grafting

The development of bone grafting can be traced back to mythology and ancient civilizations. From the biblical account of Eve being created from Adam’s rib to early forms of bone surgery observed in ancient Egyptian and Mesoamerican cultures, the idea of skeletal reconstruction has long captured human imagination (Pryor et al., 2009; Donati et al., 2007). In the 17th century, Dutch surgeon Job van Meekeren documented one of the first cases of xenogeneic bone grafting by implanting a fragment of a dog’s skull into the cranium of an injured soldier, representing an early clinical application of bone substitution (Shah et al., 2014). During the 18th and 19th centuries, the rise of scientific observation led to systematic investigations into bone regeneration. Henri-Louis Duhamel du Monceau was the first to experimentally demonstrate the osteogenic function of the periosteum (Böni, 2000). Later, Léopold Ollier and Barth conducted animal studies exploring periosteal and dead bone regeneration, providing a theoretical foundation for modern bone graft research (Kolk et al., 2012). In 1912, Vittorio Putti synthesized previous research findings and, drawing on his extensive clinical experience, proposed ten fundamental principles of bone grafting (Donati et al., 2007). These principles addressed the limited bioactivity of xenografts, the osteogenic potential of the periosteum, the importance of mechanical fixation, and the need for strict aseptic technique. Without access to modern immunology or tissue engineering, Putti established a scientific framework through careful observation and logical reasoning, marking a pivotal transition in bone grafting from empirical practice to a systematic scientific discipline.

### 4.2 The diamond concept of bone regeneration

**FIGURE 3:**
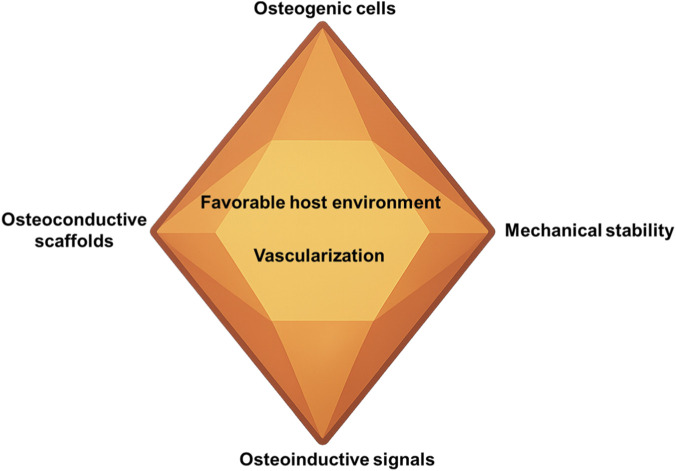
The diamond concept of bone regeneration.

The diamond concept of bone regeneration is a widely accepted framework that outlines the essential elements required for successful bone regeneration (Figure 3); (Giannoudis et al., 2007; Giannoudis et al., 2008). The model initially proposed four core components: osteogenic cells, osteoinductive signals, an osteoconductive scaffold, and mechanical stability. Osteogenic cells, including mesenchymal stem cells and periosteum-derived precursors, serve as the cellular basis for new bone formation (Marie and Cohen-Solal, 2018). Osteoinductive factors, such as bone morphogenetic proteins and platelet-derived growth factor, initiate the recruitment and differentiation of progenitor cells. Osteoconductive scaffolds provide a three-dimensional structure for cell attachment, migration, and mineralization. Mechanical stability regulates cellular behavior through the transmission of mechanical forces and stress-related signaling, ensuring structural integrity at the regeneration site.

In addition, the model has been further expanded to include two equally critical components: vascularization and a favorable host environment. A well-developed vascular network is essential for delivering oxygen, nutrients, and signaling molecules to the regeneration site (Filipowska et al., 2017). Systemic factors, such as metabolic health, immune status, and the presence of chronic diseases, also significantly influence the bone regeneration response (Gibon et al., 2017). Moreover, the concept of a sealed biological chamber emphasizes the importance of maintaining local bioactivity within a controlled microenvironment, allowing all six components to act synergistically to promote organized tissue regeneration (Calori and Giannoudis, 2011).

The diamond concept not only integrates the core biological mechanisms of bone regeneration but also provides a theoretical foundation for the rational design of bone grafts and synthetic substitutes in oral and maxillofacial applications. By defining these six critical elements, the model supports the evaluation of current materials and guides the development of next-generation grafts with improved structure, enhanced biofunctionality, and superior clinical performance. In dental applications such as alveolar ridge preservation, post-extraction socket repair, and peri-implant defect regeneration, multifactorial strategies based on this concept hold significant promise for improving regenerative outcome.

### 4.3 Essential factors in osteogenic biomaterial design

Based on the diamond concept of bone regeneration, the effective design of bone grafts and substitutes should translate the six regenerative elements into practical material characteristics, including biocompatibility, bioactivity, mechanical strength, and clinical applicability (Haugen et al., 2019; Wang and Yeung, 2017; Bhatt and Rozental, 2012; Dolcimascolo et al., 2019).

To support bone formation, materials must facilitate stem cell adhesion, proliferation, and osteogenic differentiation. This can be achieved through the incorporation of osteoinductive molecules such as bone morphogenetic proteins and platelet-derived growth factor, or by modulating the local ionic environment (Kang et al., 2025a; Liu C. et al., 2025; Shar et al., 2019; Kaigler et al., 2011). Osteoconductive capacity depends largely on scaffold architecture, particularly the presence of interconnected pores that enable cell migration, vascular penetration, and matrix deposition (Ghayor and Weber, 2018). Mechanical properties should closely match those of native bone to ensure appropriate load transmission and prevent stress shielding (Hannink and Arts, 2011; Prasadh and Wong, 2018). Degradation should be gradual and synchronized with new bone formation (Hannink and Arts, 2011).

In addition to biological functionality, graft materials should be readily available, ethically acceptable, and easy to use in surgical procedures. They should minimize immune responses, allow full integration with host bone, and eventually be replaced by regenerated tissue of similar quality and volume.

Before clinical application, candidate materials must undergo comprehensive preclinical testing. This includes *in vitro* evaluation of cytocompatibility and osteogenic potential, as well as *in vivo* studies assessing bone regeneration capacity, degradation behavior, and immune interactions. Early attention to regulatory compliance and scalable production processes is also necessary for successful clinical translation.

## 5 Bone grafts and substitutes

### 5.1 Current bone grafts and substitutes: types, advantages, and limitations

**FIGURE 4:**
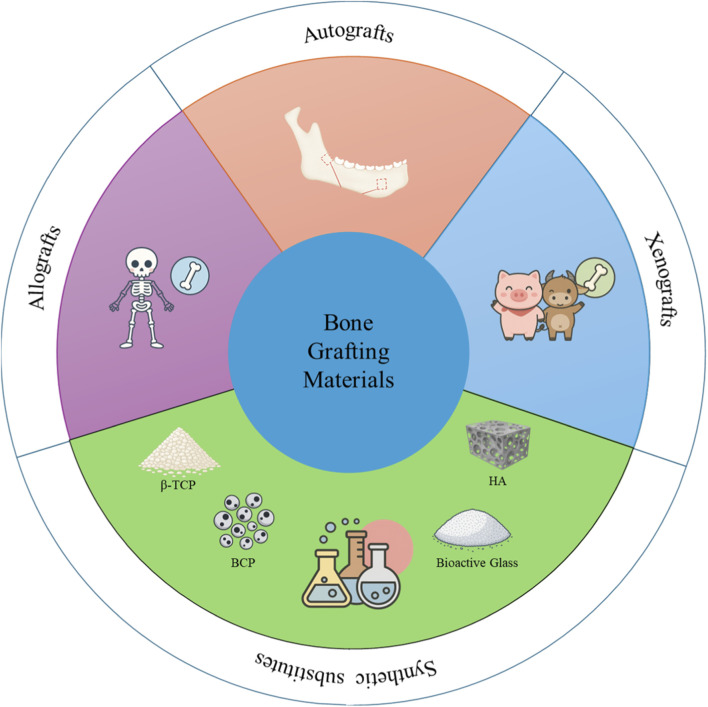
Classification of current bone grafts and substitutes.

The classification of current bone grafts and substitutes is illustrated in Figure 4. Autologous bone grafting is considered a gold standard in clinical bone regeneration (Misch, 2010; Kang et al., 2024). Common donor sites include the iliac crest, calvarium, mandible, radius, and tibia, providing both cortical and cancellous bone components. In oral and maxillofacial surgery, intraoral donor sites are particularly favored due to their surgical accessibility, minimal invasiveness, and anatomical proximity to the defect. Autografts naturally possess osteogenic, osteoconductive, and osteoinductive properties because of their viable cell content and are fully biocompatible, eliminating the risk of immune rejection (Fillingham and Jacobs, 2016). However, harvesting autologous bone requires an additional surgical site, which may cause donor site pain, bleeding, or scarring (Patel et al., 2021; Younger and Chapman, 1989). The amount of available graft material is often limited, especially from intraoral sites, and postoperative graft resorption may compromise long-term stability.

Allogeneic bone grafts are obtained from cadaveric or living donors and undergo sterilization and decellularization to ensure clinical safety (Roberts and Rosenbaum, 2012). Common forms include freeze-dried bone and demineralized bone matrix (Stavropoulos et al., 2000). These materials retain structural proteins such as type I collagen and osteoinductive molecules like bone morphogenetic proteins. They are generally osteoconductive and may have limited osteoinductive capacity, depending on processing. Allografts eliminate the need for bone harvesting from the patient and are available in larger quantities. However, they lack viable cells, which limits osteogenic potential, and carry risks such as immune response, disease transmission, and compromised mechanical strength.

Xenogeneic bone grafts, derived from non-human sources such as bovine or porcine bone, can be safely applied in humans following rigorous processing to eliminate immunogenic components (Pabst et al., 2024). Deproteinized bovine bone matrix (DBBM), such as Bio-Oss® (Geistlich, Switzerland), is the most widely used and well-documented xenograft material in clinical dentistry (Miron et al., 2000). It is composed of bovine bone granules that undergo high-temperature sintering and alkaline treatment to remove organic matter, thereby minimizing the risk of infection and immune rejection. Owing to their excellent biocompatibility, structural similarity to native bone, and accessibility, xenografts like Bio-Oss are frequently used in dental procedures such as alveolar ridge preservation and maxillary sinus augmentation, especially in settings where allografts are limited (Kang et al., 2024). Despite these advantages, xenografts tend to exhibit slow resorption rates, which may hinder timely bone remodeling. In rare instances, inadequate processing may lead to delayed integration or immunologic responses (Gultekin et al., 2016).

To address the limitations associated with biological bone grafts, various synthetic bone substitutes have been developed. These materials are primarily osteoconductive and are engineered to replicate the structural and mechanical features of native bone (Hench, 2006; Taye, 2022; Chen et al., 2020; Hong et al., 2010; Dutta et al., 1971; Shi et al., 2021). Although some exhibit limited osteointegration, they offer advantages such as ease of customization, diverse morphological forms, and elimination of donor site morbidity. This review focuses on commonly used synthetic materials in dental applications, including hydroxyapatite, β-tricalcium phosphate ceramics, biphasic calcium phosphate ceramics, and bioactive glass (Table 1).

**TABLE 1:** Clinical profiles, performance, and limitations of major synthetic and allogeneic bone substitutes.

Material	Key characteristics	Primary dental indications	Clinical performance & outcomes	Clinical limitations
Hydroxyapatite (HA)	Non-resorbable or very slow degradation; high crystallinity	• Ridge preservation (esthetic zone) • Sinus augmentation (lateral window) • Contour augmentation	• Excellent long-term volume maintenance due to slow resorption • Histological evidence of HA particles integrated with new bone after many years • High implant survival rates reported (>95%)	• Residual particles may persist permanently and potentially interfere with implant–bone contact if overfilled • Longer healing time required for formation of vital bone
β-Tricalcium Phosphate (β-TCP)	Fully resorbable; rapid degradation (≈3–12 months)	• Small periodontal defects • Cystic or tumor-related bone defects • Sinus lift (commonly mixed with autograft)	• Rapid conversion into native bone • No residual foreign material after ∼12 months • High quality of newly formed bone	• Poor volume stability; graft may resorb before complete bone maturation • Risk of soft-tissue collapse in large defects
Biphasic Calcium phosphate (BCP)	Balanced resorption (HA for stability, β-TCP for turnover)	• Sinus floor elevation • Large bony defects • Peri-implant defects	• Predictable regeneration: early β-TCP resorption with long-term HA space maintenance • Widely accepted synthetic standard for sinus augmentation with outcomes comparable to xenografts	• Clinical performance highly dependent on HA/β-TCP ratio • Initial mechanical strength lower than autogenous bone
Bioactive glass	Surface reaction forms hydroxycarbonate apatite; high bioactivity	• Periodontal intrabony defects • Cystic cavities • Ridge preservation	• Rapid bonding to bone and soft tissue • Reported antibacterial effects beneficial in infected sites • High proportion of vital bone formation	• Handling difficulties (loose granules; often requires a binder) • Resorption rate may be unpredictable depending on formulation
Demineralized bone matrix (DBM)	Allogeneic; collagen matrix containing growth factors (e.g., BMPs)	• Periodontal defects • Adjunct (“booster”) combined with other grafts • Non-load-bearing defects	• Osteoinductive potential may accelerate early healing • Good biological integration, primarily serving as a bioactive scaffold	• Limited structural stability (putty or paste form) • Batch-to-batch variability in BMP content • Ethical and regulatory concerns related to human origin

Despite the widespread clinical use of these materials, challenges persist. These include limited osteoinductive performance, variability between patients, and incomplete graft integration. Personalized treatment strategies are receiving increasing attention. Three-dimensional (3D) printing and computer-aided design/manufacturing (CAD/CAM) technologies have been applied to precisely fabricate allogeneic bone blocks that match the exact geometry of the defect, demonstrating high accuracy and favorable clinical outcomes in complex reconstructions such as alveolar ridge augmentation. These approaches eliminate the need for autologous bone harvesting and manual modification of the graft, thereby reducing surgical time, minimizing the risk of contamination and complications, and maximizing the contact area between the bone block and the recipient site through precise adaptation, which promotes optimal graft integration (Kim et al., 2024; Brachet et al., 2023).

Research is also focusing on improving the biological activity of graft materials. Incorporating mesenchymal stem cells (MSCs), bone morphogenetic proteins (BMPs), platelet-derived growth factor (PDGF), and insulin-like growth factor (IGF) have demonstrated osteoinductive potential and can accelerate bone regeneration in defect sites (Oliveira et al., 2021; Shang et al., 2021; Blokhuis, 2009). However, their clinical use still requires careful assessment of dosage, safety, and delivery efficiency. Ongoing research is expected to lead to more advanced, adaptable, and biologically responsive graft materials for complex bone reconstruction.

### 5.2 Hydroxyapatite

Hydroxyapatite (HA), with the molecular formula Ca_10_(PO_4_)_6_(OH)_2_, is a naturally occurring form of calcium phosphate and constitutes the principal inorganic phase in human bone and dental tissues (Shi et al., 2021). Because of its compositional similarity to biological apatite, HA demonstrates high biocompatibility, intrinsic bioactivity, osteoconductivity, and low immunogenicity, which makes it widely applicable in orthopedic and dental regenerative therapies. (Dutta et al., 1971; Mondal et al., 2023; Meng et al., 2022).

Natural HA typically displays a porous microarchitecture, with porosity ranging between 60 and 70 percent and pore diameters spanning 50–200 μm (Bertocco et al., 2025). This porous network facilitates cell attachment, migration, and the infiltration of blood vessels, thereby supporting bone tissue ingrowth. Owing to its slow degradation rate *in vivo*, HA can provide extended mechanical stability during the early stages of new bone formation. The flexural strength of pure hydroxyapatite typically ranges from 38 to 250 MPa, with a compressive strength of 120–150 MPa and a tensile strength of 38–300 MPa, indicating that it is a typical brittle material best suited for the repair of non-load-bearing sites and small bone defects (Liu W. et al., 2025; Heimbach et al., 2018).

To overcome these limitations, various composite strategies have been developed. For example, combining HA with tricalcium phosphate (TCP) can accelerate resorption and promote balanced bone remodeling; incorporating collagen can improve mechanical toughness and enhance osteoblast differentiation; integrating HA with biodegradable polymers such as poly (lactic-co-glycolic acid) (PLGA) or polycaprolactone (PCL) can enhance structural performance and facilitate progenitor cell adhesion (Hai et al., 2014; Jiao et al., 2019).

In addition, nanoscale modifications of HA have attracted considerable attention. Doping with metallic ions such as strontium (Sr^2+^), magnesium (Mg^2+^), or zinc (Zn^2+^) can improve osteoinductive potential while imparting antibacterial properties (Kubiak-Mihkelsoo et al., 2025). Emerging fabrication techniques, including 3D printing of customized HA scaffolds and injectable nano-HA formulations, have shown promise in modulating immune responses and directing stem cell differentiation toward osteogenic lineages (Babilotte et al., 2021; Wang F. et al., 2022).

Despite these advancements, challenges remain regarding the clinical translation of HA-based biomaterials. These include achieving an optimal balance between porosity and mechanical strength, fine-tuning degradation rates, and validating the safety and efficacy of these materials in long-term clinical settings. Future research should focus on developing multifunctional and patient-specific HA constructs, aiming to expand their utility in advanced bone tissue engineering applications.

From a clinical standpoint, a recent systematic review further validated the utility of hydroxyapatite in alveolar ridge preservation (ARP). The analysis concluded that the application of HA grafts in extraction sockets significantly minimizes both horizontal and vertical ridge resorption compared to spontaneous healing. By effectively maintaining ridge dimensions, HA reduces the need for additional augmentation procedures during subsequent implant placement, although its slow resorption rate implies that residual particles often integrate with the newly formed bone (Razali et al., 2024).

### 5.3 β-tricalcium phosphate ceramics

β-Tricalcium phosphate (β-TCP, Ca_3_(PO_4_)_2_) is a widely used synthetic calcium phosphate bone substitute characterized by excellent biocompatibility, resorbability, and moderate osteoconductive capacity (Jeong et al., 2019; Saito et al., 2024). Unlike HA, β-TCP possesses excellent degradability and can be gradually resorbed by osteoclasts after implantation, subsequently being replaced by newly formed bone tissue (Bohner et al., 2020). Its porous structure typically contains macropores (100–600 µm) and micropores (0.1–5 µm), which facilitate osteogenic cell infiltration, neovascularization, and nutrient exchange. These characteristics endow β-TCP with excellent osteoconductive and angiogenic properties, playing a vital role in the bone healing process (Mayr et al., 2013). Furthermore, β-TCP plays an osteoinductive role through the local release of calcium and phosphate ions, which promote the differentiation of stem cells into osteoblasts (Nawa et al., 2024).

The clinical use of β-TCP in maxillofacial surgery is well established. In dental practice, it is commonly applied for alveolar ridge augmentation and maxillary sinus floor elevation in association with implant placement (Okada et al., 2016; Daher et al., 2019). Typically, β-TCP granules can complete the resorption and integration process within 3–6 months after implantation (Garcia et al., 2022). Although β-TCP provides improved mechanical properties compared to many other synthetic grafts, it still has lower mechanical strength than natural cancellous bone and allografts, which limits its use to regions with low or moderate mechanical load. Nonetheless, its biological performance can be enhanced by optimizing porosity, surface morphology, and pore orientation to improve cellular attachment, vascular ingrowth, and tissue integration (Putri et al., 2022).

In recent years, the osteogenic and angiogenic capabilities of β-tricalcium phosphate (β-TCP) have been substantially improved by incorporating trace elements naturally found in the body, such as zinc (Zn), silicon (Si), magnesium (Mg), and strontium (Sr) (Saito et al., 2024). These elements contribute synergistically to bone regeneration: Zn promotes osteoblastic activity and suppresses osteoclastic bone resorption, Si facilitates osteogenic differentiation and mineralization, and Mg enhances biocompatibility while modulating bone matrix metabolism due to its bone-mimicking elastic modulus (Kang et al., 2025b). Formulations containing MgO, ZnO, SrO, and SiO_2_ have been shown to significantly improve the osteogenic differentiation of bone marrow mesenchymal stem cells (Saito et al., 2024). Coupled with fabrication technologies such as 3D printing and foam replication, these advances allow β-TCP scaffolds to be tailored to patient-specific needs (Nandi et al., 2018). With this combination of osteoconductivity, biodegradability, and structural customizability, β-TCP continues to show strong potential in bone regeneration and reconstructive applications. Ongoing innovations in material functionalization are expected to further broaden its clinical utility.

From a clinical perspective, systematic evaluations have substantiated the efficacy of β-TCP in implant dentistry. Analysis of data from multiple randomized clinical trials indicates that the survival rates of dental implants placed simultaneously with β-TCP grafting are comparable to those placed with other standard bone substitutes or in native bone (Roca-Millan et al., 2022). These findings confirm that β-TCP is a reliable synthetic alternative for bone augmentation procedures, particularly in cases where avoiding animal-derived grafts is preferred, although further long-term histological evidence is recommended to fully predict its resorption dynamics in large defects.

### 5.4 Biphasic calcium phosphate ceramics

Biphasic calcium phosphate (BCP) ceramics, composed of hydroxyapatite (HA) and β-tricalcium phosphate (β-TCP), combine the distinct advantages of both components (Liu W. et al., 2025). HA shares chemical similarity with natural bone, its physical properties differ significantly. Its elastic modulus and compressive strength are generally much higher than those of native bone, which may theoretically lead to stress shielding and subsequent resorption of surrounding bone tissue. To address this limitation, β-tricalcium phosphate (β-TCP), which has a lower compressive strength and a faster degradation rate, is incorporated to enhance osteoconductivity and promote new bone formation (Chen et al., 2020; Owen et al., 2018). By adjusting the HA/β-TCP ratio and optimizing the microporosity, macroporosity, and interconnectivity, the dynamic balance between material degradation and osteogenic performance can be precisely regulated. This allows BCP to offer sustained mechanical support during healing while promoting cellular and vascular infiltration, thereby improving bone integration (Daculsi et al., 1997; Bouler et al., 2017).

The porous architecture of BCP and its tunable pore orientation further enhance its biological performance. These features make it suitable for repairing bone defects in low to moderate load-bearing areas. In addition, BCP can be functionalized through the incorporation of bioactive ions such as strontium or magnesium (Pina et al., 2010), or by loading osteoinductive molecules including bone morphogenetic proteins and collagen. Such modifications improve its regenerative capacity and cellular responsiveness. The use of advanced manufacturing techniques, including three-dimensional printing and foam replication, enables the fabrication of customized BCP scaffolds with patient-specific geometry and tailored functional properties.

Biphasic calcium phosphate (BCP) ceramics exhibit excellent biocompatibility, osteoconductivity, and osteoinductivity (Hong et al., 2010; Chen Y. et al., 2014; Tian et al., 2017). These intrinsic biological properties, combined with BCP’s compositional tunability, controlled biodegradability, and favorable mechanical strength, make it a versatile and clinically promising platform for bone tissue engineering. Furthermore, its potential for surface biofunctionalization enhances its suitability for reconstructive procedures that demand both structural integrity and biological performance.

### 5.5 Bioactive glass

Bioactive glass was first developed in 1969 by the Larry L. Hench research group, leading to the formulation of the well-known 45S5 composition (Hench, 2006). This material primarily consists of approximately 45 percent silica, along with calcium oxide, sodium oxide, and phosphorus pentoxide (Dash et al., 2023). Upon implantation, its surface undergoes a rapid reaction with body fluids, initially forming a silica-rich gel layer that subsequently mineralizes into a hydroxycarbonate apatite phase within hours to days (Soltani and Alizadeh, 2022). This bioactive layer supports strong bonding with surrounding bone tissue and facilitates osteointegration.

The material demonstrates excellent biocompatibility and osteoconductivity (Krishnan and Lakshmi, 2013). Its porous structure enhances resorption and allows for efficient infiltration of bone-forming cells and blood vessels (Chand et al., 2024). Beyond its structural role, the release of ionic species such as calcium, phosphate, and silicon promotes osteoblastic activity and bone regeneration (Naseri et al., 2017). Certain formulations, such as S53P4, have been shown to inhibit the growth of oral pathogens, thereby providing both regenerative and antibacterial functions specifically relevant to dental and maxillofacial applications (Stoor et al., 1998; Hu et al., 2009; Begum et al., 2016).

Despite these advantages, the inherent brittleness and low fracture toughness of bioactive glass restrict its use in load-bearing applications (Chen et al., 2006; Chen Q. et al., 2014). To overcome these limitations, it is often combined with polymers or metals, or used in the reconstruction of small, non-load-bearing defects (Pant et al., 2022; Kazemi et al., 2018; Zhu et al., 2012; Wang C. et al., 2022; Jimén et al., 2020; Zheng et al., 2021). Recent developments have introduced borate- and phosphate-based bioactive glasses, which offer improved compositional tunability and more predictable degradation rates (Abodunrin et al., 2023; Fakher and Westenberg, 2024; Ege et al., 2022; Christie et al., 2017; Kowalska et al., 2024). In addition, innovations such as nanoscale fabrication, mesoporous structural design, and ionic doping have significantly enhanced the material’s osteoinductive and angiogenic potential (Taye, 2022; Zheng et al., 2021).

Overall, bioactive glass is regarded as a multifunctional and highly versatile material. Its controlled degradability, structural modifiability, and biological responsiveness make it a promising candidate for a wide range of applications in bone tissue engineering and clinical bone regeneration.

From a clinical standpoint, a recent systematic review and meta-analysis provided compelling evidence regarding the dimensional stability of bioactive glass in maxillofacial reconstruction. The study revealed that bioactive glass grafts demonstrated significantly greater total bone volume retention and a lower resorption rate at 6 months post-surgery compared to autogenous bone grafts while achieving comparable levels of new bone formation (Saghiri et al., 2025). These findings suggest that bioactive glass serves as a durable alternative for reconstructive procedures where long-term graft volume maintenance is critical, effectively overcoming the unpredictable resorption often associated with autografts.

### 5.6 Demineralized bone matrix (DBM)

Demineralized bone matrix (DBM) is a form of allogeneic graft material characterized by the removal of its mineral content (Gruskin et al., 2012). This process preserves type I collagen and osteoinductive growth factors, including varying concentrations of bone morphogenetic proteins (BMPs) and potentially transforming growth factors (TGFs), which collectively confer strong osteoinductive capacity to DBM (Kim et al., 2025). Compared to mineralized allografts, including cortical and cancellous bone, DBM demonstrates superior ability to induce new bone formation. However, DBM itself lacks osteogenic capacity because it does not contain viable cells capable of directly generating new bone (Chen et al., 2021). Instead, it promotes bone regeneration by slowly releasing signaling molecules that recruit and stimulate host cells (Gruskin et al., 2012).

Traditional DBM is challenging to handle due to its friable texture, lack of malleability, and tendency to disperse in bleeding surgical fields (Jang et al., 2008). DBM often requires other materials as carriers, such as chitosan, hydroxyapatite (HA), calcium sulfate (CS), alginate, carboxymethyl cellulose (CMC), and type I collagen (Chen et al., 2021; Zhang et al., 2019). These carriers improve moldability and handling characteristics but may dilute the concentration of active components, potentially diminishing osteoinductive efficacy.

To minimize the risks of immune reactions, allergic responses, and transmissible diseases such as spongiform encephalopathy, effective sterilization of DBM is essential prior to clinical use (Titsinides et al., 2019). Traditional sterilization methods include gamma irradiation, electron beam, and ethylene oxide (ETO) treatment. While these approaches are effective in microbial decontamination, high-temperature processing or the use of chemical sterilants such as formaldehyde and glutaraldehyde can denature bone morphogenetic proteins (BMP), thereby diminishing the osteoinductive capacity and clinical efficacy of DBM (Gruskin et al., 2012).

As a result, many manufacturers have adopted aseptic processing as an alternative strategy. This method is carried out in controlled cleanroom environments using standardized procedures to minimize the risk of microbial contamination. Freeze-drying and cold storage are widely recognized as the most effective approaches for preserving the structural integrity and long-term biological activity of DBM (Gruskin et al., 2012).

## 6 Discussion and future perspectives

Bone defects can result from various causes, including trauma, tumor resection, degenerative diseases, congenital malformations, and dental procedures. To address these conditions, more than 2.2 million bone grafting procedures are performed each year across orthopedic, neurosurgical, and dental specialties worldwide. In recent years, the increasing demand for craniofacial reconstruction and the widespread use of dental implants have significantly expanded the application of bone grafts and substitutes in dentistry (Zhao et al., 2021).

The clinical practice of bone grafting dates back to the 17th century, when Dutch surgeon Job van Meekeren documented one of the earliest recorded xenogeneic grafts by implanting a fragment of a dog’s skull into a human cranial defect (Shah et al., 2014). The integration of materials science with surgical techniques has since driven the advancement of novel bone graft materials, offering new solutions for bone regeneration and the repair of complex skeletal defects.

Although the criteria for an ideal bone graft material were established decades ago, autologous bone remains the gold standard in clinical practice due to its osteogenic, osteoinductive, and osteoconductive properties, and its lack of immunogenicity and disease transmission risk (Dewi and Ana, 2018). However, donor site morbidity and limited availability have prompted the exploration of alternative graft materials. Allografts and xenografts have demonstrated favorable outcomes but still raise concerns regarding immune reactions and potential pathogen transmission (Koons et al., 2020). Synthetic bone substitutes have been developed to overcome these limitations and offer several advantages, such as consistent availability and the potential for customized manufacturing to match the geometry of specific bone defects, facilitating personalized treatment (Zhang et al., 2025).

Patient personal beliefs, such as religious faith or ethical veganism, significantly influence the acceptance of bone grafting materials (Assari et al., 2022). Many patients refuse xenogeneic or allogeneic materials due to moral reasons, sometimes even opting to forego treatment. This shift in values not only requires clinicians to fully respect patient autonomy when formulating treatment plans but also directly drives market demand and research for animal-free “vegan-friendly” fully synthetic materials, making them an important clinical choice for specific patient groups (Wang et al., 2025).

Despite these advantages, synthetic materials often lack the mechanical strength of native bone and present challenges in balancing biocompatibility with controlled degradation. Moreover, when combined with biologics such as growth factors or stem cells, questions remain about their long-term stability, sustained bioactivity, and *in vivo* safety. Current research efforts focus on developing multifunctional and biomimetic graft materials (Oliveira et al., 2021; Shang et al., 2021). Strategies include incorporating osteoinductive agents and mesenchymal stem cells to enhance regenerative capacity, and engineering scaffolds with controlled release capabilities to maintain the bioactivity of therapeutic molecules over an extended period. In parallel, structural design has become increasingly important, with attention directed toward optimizing pore size, morphology, interconnectivity, density, and degradation rate to better replicate the native bone environment and support cell adhesion, migration, and vascularization.

However, the introduction of such functionalization strategies presents complex regulatory challenges. Currently, most bone graft materials are classified as Class II medical devices (e.g., via the “substantial equivalence” pathway in the US), which involves relatively streamlined approval but still requires rigorous data on biocompatibility, sterility, and manufacturing consistency (Gillman and Jayasuriya, 2021). However, with the introduction of functionalization strategies like growth factors or stem cells, novel materials are often reclassified as combination products or high-risk devices, necessitating stricter clinical trials and long-term assessment. While this ensures safety, it significantly increases development costs and delays the translation cycle. Therefore, incorporating regulatory compliance into the early stages of material design has become a critical step in facilitating clinical translation.

From a clinical perspective, the most significant bottleneck restricting the widespread application of emerging synthetic materials is the scarcity of long-term evidence compared to bovine-derived xenografts. While xenografts are associated with ethical and immunogenic concerns, they possess decades of validated efficacy and predictability. In contrast, although synthetic materials (such as calcium phosphate ceramics) offer advantages in biological safety and show good short-term osteogenic potential, their long-term (5–10 years) behavior regarding resorption, volume maintenance, and delayed complications remains uncertain. This “evidence gap” forces clinicians to balance “time-tested reliability” with “engineered safety,” often leading to conservative choices in complex cases. Thus, only by filling this void through standardized, long-term longitudinal studies can clinical confidence be truly established, driving new materials from “cautious trials” to routine application.

To achieve this, comprehensive research systems are needed that go beyond preclinical models. Future efforts must integrate long-term follow-up and mechanistic studies to thoroughly evaluate the safety and efficacy of emerging materials, thereby facilitating their commercialization and clinical adoption. Overall, while this review highlights meaningful advances in the field of dental bone grafting, continued interdisciplinary collaboration is essential to develop materials that combine porosity, mechanical integrity, controllable resorption, and remodeling properties aligned with the pace of natural bone regeneration.
